# Fluorescent nanosensors for intracellular measurements: synthesis, characterization, calibration, and measurement

**DOI:** 10.3389/fphys.2013.00401

**Published:** 2014-01-16

**Authors:** Arpan S. Desai, Veeren M. Chauhan, Angus P. R. Johnston, Tim Esler, Jonathan W. Aylott

**Affiliations:** ^1^Laboratory of Biophysics and Surface Analysis, School of Pharmacy, University of NottinghamNottingham, UK; ^2^Department of Chemical and Biomolecular Engineering, The University of MelbourneVictoria, Australia

**Keywords:** nanosensor, fluorophore, inert matrix, transducer, polyacrylamide, silica sol-gel, cellular delivery, uptake

## Abstract

Measurement of intracellular acidification is important for understanding fundamental biological pathways as well as developing effective therapeutic strategies. Fluorescent pH nanosensors are an enabling technology for real-time monitoring of intracellular acidification. The physicochemical characteristics of nanosensors can be engineered to target specific cellular compartments and respond to external stimuli. Therefore, nanosensors represent a versatile approach for probing biological pathways inside cells. The fundamental components of nanosensors comprise a pH-sensitive fluorophore (signal transducer) and a pH-insensitive reference fluorophore (internal standard) immobilized in an inert non-toxic matrix. The inert matrix prevents interference of cellular components with the sensing elements as well as minimizing potentially harmful effects of some fluorophores on cell function. Fluorescent nanosensors are synthesized using standard laboratory equipment and are detectable by non-invasive widely accessible imaging techniques. The outcomes of studies employing this technology are dependent on reliable methodology for performing measurements. In particular, special consideration must be given to conditions for sensor calibration, uptake conditions and parameters for image analysis. We describe procedures for: (1) synthesis and characterization of polyacrylamide and silica based nanosensors, (2) nanosensor calibration and (3) performing measurements using fluorescence microscopy.

## Introduction

### Background

Fluorescent nanosensors are powerful tools, which represent an advance in sensor-based technologies. Due to their size, inert matrix, signal intensity and ratiometric properties they can be utilized to accurately characterize sub-cellular compartments and make real-time measurements in microenvironments of interest (Aylott, 2003).

Earlier work within this field focused on reducing the dimensions of the sensing elements in conventional sensors, such as microelectrodes (Menon and Martin, 1995) and fiber optic sensors (Shortreed et al., 1996; Song et al., 1997; Ruckruh et al., 1999). For fiber optic sensors the sensing element is often found at the distal tip of the optode, which commonly contains an analyte responsive fluorophore. Using laser heated optical fiber pulling techniques; tip dimensions of less than 50 nm diameter have been reported for cellular insertion (Vo-Dinh, 2003). When coupled with established detection systems, such as fluorescence and confocal microscopy, calibrated pulled optical fibers can be used to characterize local changes in fluorescence and in turn analyte concentrations in biological systems.

The major drawback of this method is the substantial damage which can be caused when fiber optic tips are inserted into biological systems, especially with regard to cells (Monson et al., 2003). Cellular perturbations have been attributed to the initial puncture of the cell membrane as well as the volume the optode occupies within the cell (Clark et al., 1998).

Several alternative approaches to intracellular pH measurement have been proposed including surface enhanced Raman scattering (SERS) based sensors (Kneipp et al., 2010), green florescent protein (GFP) based sensors (Kneen et al., 1998), and RNA based sensors (Paige et al., 2012). However, the most widely implemented approach utilizes pH-sensitive fluorophores. In general, fluorophores generate a fast, bright response, which can also be quantified by fluorescence microscopy. These properties make them ideal candidates for rapid, real-time measurements in cells (Resch-Genger et al., 2008). However, their use in a free form for making quantitative measurements is limited due to the difficulty associated with cellular delivery (Webster et al., 2007), interference from cellular components and non-ratiometric measurements (Xu et al., 2002).

A number of fluorophores are commercially available which have been chemically modified to enhance delivery e.g., acetoxymethyl and acetate esters (Han and Burgess, 2009). Although chemical modification has demonstrated improved delivery, it is not always possible to engineer fluorophores in this way without affecting their sensing capabilities. Furthermore, free fluorophores have also been found to interact with cellular components. Interaction with cellular components can hinder sensing capabilities and/or initiate cellular toxicity. Sensing capabilities can be affected as a result of protein binding (Graber et al., 1986), leading to fluorescence quenching and inaccurate measurements. While cellular toxicity could arise from photo excitation of fluorophores (Srivastava et al., 2007).

Ratiometric fluorophores enhance the accuracy of measurements made. This is achieved through the elimination of interference caused by; fluctuations in excitation source, detector sensitivity, light scattering and fluorophore concentration (Park et al., 2005). It is important to note very few fluorophores are intrinsically ratiometric. In addition, the delivery of a secondary reference fluorophore will not necessarily produce ratiometric measurements. This is because different fluorophores can be found at different cellular locations and concentrations, which may also interfere with biological components and will result in erroneous measurements.

Fluorescent nanosensors combine the benefits of conventional sensors, whilst overcoming some of their inherent weaknesses. They are spherical particles, of ~30–500 nm in diameter. Due to their small size, in comparison to the total volume of pulled optical fibers, fluorescent nanosensors boast a high surface/volume ratio (Clark et al., 1998). This means, when imaged using fluorescence or confocal microscopy, fluorescent nanosensors can be delivered in high quantities with minimal cell perturbations (Clark et al., 1999), producing high resolution images (Schulz et al., 2010).

Fluorescent nanosensors are composed of an inert matrix, such as polyacrylamide or silica sol-gel, which entraps or is covalently bound to fluorophores. The nanoparticle matrix shields the sensing elements from external biological interferants as well as protecting cellular components from potentially harmful fluorophores.

**Figure 1:**
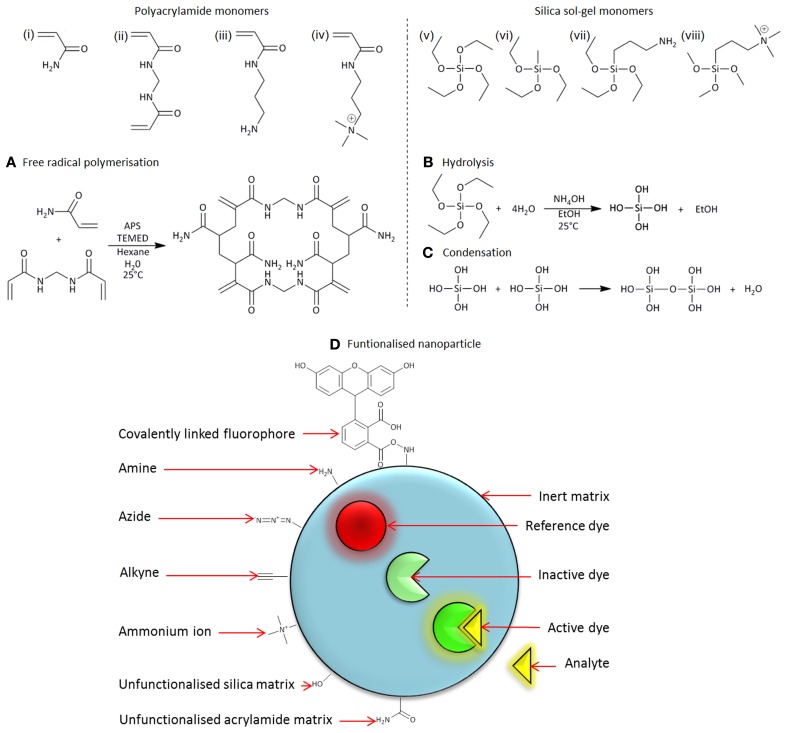
**Monomers (i–iv) and (v–viii) are used to synthesize polyacrylamide and silica sol-gel nanoparticles, respectively: (i) acrylamide, (ii) N,N′-methylenebis(acrylamide), (iii) N-(3-aminopropyl)methacrylamide (APMA), (iv) (3-acryamidopropyl)trimethylammonium (ACTA), (v) tetraethoxysilane (TEOS), (vi) methyltriethoxysilane (MTEOS), (vii) (3-aminopropyl) triethoxysilane, and (viii)-trimethoxysilylpropyl-N,N,N- trimethyl-aminium (TMAC). (A)** Free radical polymerization of polyacrylamide monomers. **(B)** Hydrolysis and **(C)** condensation of silica sol-gel monomers. **(D)** Diagrammatic representation of a functionalized nanoparticle.

Nanoparticles can be loaded with high numbers of sensing elements, therefore improving their signal/background ratio when imaged. Typically, fluorescent nanosensors consist of two types of fluorophore; an indicator and a reference, Figure 1D. The indicator functions as a transducer, which produces a signal corresponding to the concentration of the analyte of interest. In contrast, the reference fluorophore is insensitive to changes in analyte concentration, producing a constant signal at a wavelength different to the indicator fluorophore. The combination of indicator and reference fluorophores permits accurate ratiometric measurements to be made. Furthermore, because the matrix permits inclusion of more than one type of fluorophore, there is scope for simultaneous measurement of two or more parameters with the same nanosensor. Examples, of the types of fluorophores, which have been used in fluorescent nanosensors, can be found in Table 1 (list of indicator and reference dyes).

**Table 1:** **Selected commercially available indicator and reference fluorophores**.

**Analyte**	**Fluorophore**	**λ_max,abs_ (nm)**	**λ_max,em_ (nm)**
pH (near neutral)	BCECF	503	525
	BCPCF	505	527
	Carboxyfluorescein	492	516
	CarboxySNARF-1	544	575
pH (acidic)	Oregon green 488	490	514
	CDCF	503	525
	HPTS	405	514
	Acridine orange	495	530
Oxygen	Ru(II)-tris(4,7-diphenyl-1,10-phenanthroline) chloride	455	615
Reference	Alexa 488	495	519
	TAMRA	555	580

The majority of fluorophores are available in an amine reactive form.

Abbreviations: BCECF, 2′,7′-Bis-(2-carboxyethyl)-5-(and-6-)carboxyfluorescein; BCPCF, 20,70-bis-(2-carboxypropyl)-5-(and 6)carboxyfluorescein; CDCF, 5(6)- carboxydichlorofluorescein; HPTS, 8-hydroxypyrene-1,3,6-trisulfonic acid; TAMRA, 6-carboxytetramethylrhodamine.

The majority of fluorescent nanosensors incorporate a single commercially available pH sensitive fluorophore and a separate pH insensitive reference fluorophore. On their own, commercially available pH sensitive fluorophores are only able to measure part of the intracellular pH range. Incorporation of multiple pH sensitive fluorophores in the nanosensor matrix has resulted in a sensor design suitable for pH measurement across the entire physiological pH range (Chauhan et al., 2011; Sun et al., 2011). This sensor design incorporates two pH sensitive fluorophores with identical emission spectra but different p*K*_*a*_ values (5(6)-FAM p*K*_*a*_ 6.5, Oregon Green, p*K*_*a*_ 4.8) and a reference fluorophore TAMRA. Oregon Green is optimally responsive in the acidic range (~3.5–5.5) whilst 5(6)-FAM is optimally responsive in the near neutral range (~5.5–7.5). Consequently at the intracellular acidic extreme, pH 4.0, the fluorescent nanosensor is responsive to change in pH due to Oregon Green; whereas 5(6)-FAM is effectively optically silent. As the pH increases toward near-neutral the responsiveness of Oregon Green diminishes and the responsiveness of 5(6)-FAM increases. The net result is that the overall response of the nanosensor is maintained. In this way sensors can be generated with a pH measurement range between 3.5 and 7.5.

### Polyacrylamide based nanosensors

Polyacrylamide is a common type of matrix used for nanoparticle synthesis. Polyacrylamide is inert, hydrophilic, porous, and inexpensive to produce with standard laboratory equipment (Aylott, 2003). These properties make polyacrylamide a model matrix suitable for biological applications.

**Figure 2:**
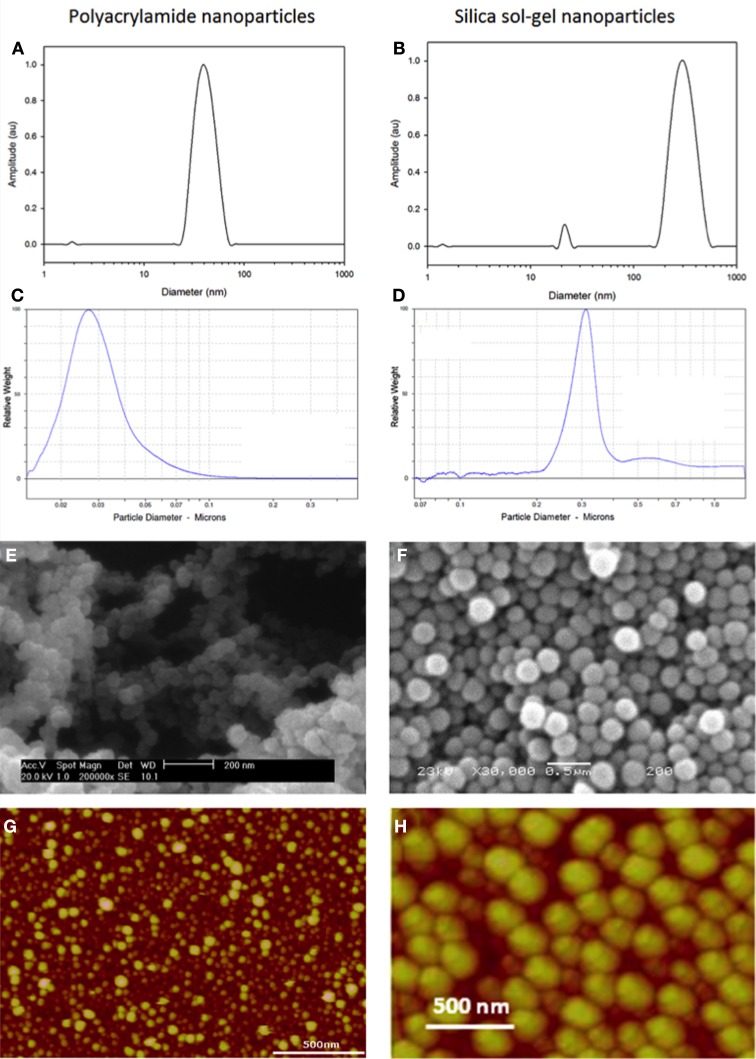
**Typical dynamic light scattering (A,B), disc centrifuge (C,D), scanning electron microscopy (E,F) and atomic force microscopy data (G,H) for polyacrylamide and silica sol-gel nanoparticles**.

Polyacrylamide nanoparticles have a size ranging between 30 and 100 nm in diameter (Figures 2A,C,E,G). They are composed of acrylamide (Figure 1i) and a cross linker, N,N′methylene-bisacrylamide (Figure 1ii), which have been polymerized in the aqueous phase of an inverse water-in-oil microemulsion (Figure 1A).

The inverse microemulsion comprises a continuous hexane hydrophobic phase (oil) and a hydrophilic aqueous phase (water). The interfaces of the inverse microemulsion are stabilized with non-ionic and anionic surfactants, polyoxyethylene(4) lauryl ether (Brij 30^®^) and dioctyl sulfosuccinate sodium (AOT), respectively. Through careful control of the water, oil and surfactant ratio a narrow distribution of nano-sized water droplets are created. The size of the water droplet directly affects the size of the nanoparticles produced. This is because acrylamide monomers are subjected to free radical polymerization in the water droplet. The polymerization is accelerated with the use of a redox pair catalyst, ammonium persulfate (APS) and N,N,N′,N′-tetramethylethylenediamine (TEMED). This article will describe a method for the synthesis of polyacrylamide nanoparticles, of ~40 nm in diameter, which can be used to trap or covalently attach fluorophores to the matrix.

### Silica sol-gel based nanosensors

Silica sol-gel, like polyacrylamide, is inert, hydrophilic, porous, and inexpensive to produce. Silica sol-gel is also transparent, photo and thermo stable (Aylott, 2003). These properties make silica sol-gel an ideal matrix for quantitative spectrophotometric measurements.

Silica sol-gel nanoparticles have a size distribution centered between 300 and 500 nm diameter (Figures 2B,D,F,H). They are composed of orthosilicate substituted monomers, such as tetraethyl orthosilicate (TEOS) (Figure 1v) and methyltriethoxysilane (Figure 1vi), which have undergone a hydrolysis and condensation reaction to produce a three-dimensional matrix. During the hydrolysis phase alkoxide groups are substituted with hydroxyl groups, forming silanols, Figure 1B. Condensation of silanol groups forms the backbone of the silica matrix, the siloxane bond, Figure 1C. The size and morphology of the silica nanoparticles is dependent on the water/monomer ratio (*r*-value), mode of catalysis and the hydrolysis time. The *r*-value influences hydrolysis by influencing the degree of alkoxide group substitution. Using a molar excess of water, a high *r*-value, favors the formation of Si(OH)_4_(Xu et al., 2001). Highly hydroxyl substituted monomers, such as Si(OH)_4_, encourage the formation of a compact silica matrix.

Hydrolysis of the monomers can be acid or base catalyzed; the type of catalysis used determines the overall morphology of the silica construct. Acid catalyzed hydrolysis at pH less than 2, results in the formation of a two dimensional mesh matrix. Whereas, base catalyzed hydrolysis at pH greater than 7, produces spherical three dimensional matrices (Burns et al., 2006a). The hydrolysis time has been shown to affect particle size (Koo et al., 2004). The particle size is inversely proportional to hydrolysis time, therefore, the greater the hydrolysis time the smaller the particle. Through manipulation of the rate of monomer addition the hydrolysis time and particle size can be controlled. Bearing the above in mind, the methods outlined in this article use an *r*-value greater than 50, a base with a pH greater than 8 and controlled rate of monomer addition (50 μl/min), to synthesize particles of ~300 to 500 nm in diameter.

### Chemical tailoring of nanosensors

Polyacrylamide and silica sol-gel nanoparticles are constructed from an architecture, which can be chemically tailored. Chemical tailoring, in essence, is the functionalization of the nanoparticle matrix, (Figure 1D), which is important for two main reasons (1) for the incorporation of fluorophores into the sensor, and (2) to control intracellular delivery.

A useful type of chemical tailoring for nanosensors is amine functionalization. For polyacrylamide and silica sol-gel nanoparticles this is achieved through the incorporation of N-(3-aminopropyl)-methacrylamide (APMA) (Figure 1iii) (Sun et al., 2009) and (3-aminopropyl)-triethoxysilane (APTES) (Figure 1vii) (Peng et al., 2007; Murdock et al., 2008), into the nanosensor matrix, respectively. Amino groups provide a site for covalent attachment of fluorophores, many of which are commercially available in an amine-reactive form. This is the most efficient method for incorporating fluorophores into the matrix without leaching. Nanosensors, that exhibit leaching of fluorophores, are compromised and unable to make accurate measurements, because fluctuations in fluorescence can be attributed to signals from both nanosensors and leached fluorophores. Alternatively, fluorophores can be incorporated into nanosensors by entrapment. By this method the fluorophore is attached to an inert molecule (e.g., 10,000 mW dextran), which is large enough to be trapped into the matrix of the sensor. This is advantageous in scenarios where conjugation is not possible, or results in disruption of fluorophore performance.

Chemical tailoring is also important for controlling intracellular delivery. Polyacrylamide and silica nanosensors are not efficiently taken up into cells when simply incubated in the culture medium and require additional methods to facilitate delivery. Nanosensors can be introduced into a cell by physical methods such as gene gun and picoinjection. Alternatively the properties of the sensor can be chemically tailored to facilitate uptake by endocytosis, this can be achieved either through conjugation of a secondary precursor such as a cell penetrating peptide (Coupland et al., 2009) or altering the physicochemical characteristics of the sensor to promote uptake (Sun et al., 2009). The advantage of delivering nanosensors by endocytosis is that it is less invasive; however it is also more challenging to control the intracellular location. The method of delivery is ultimately dependent on the aims of the study. In this article, we focus on delivering nanosensors using chemical tailoring by altering the surface charge of the sensor to induce uptake.

The surface charge of a nanosensor can be determined by measurement of Zeta potential, which is the potential difference between the stationary ions surrounding the nanoparticle and the ions in the suspending media and is strongly linked to the cellular uptake of nanoparticles (Dausend et al., 2008; Harush-Frenkel et al., 2008; Chen et al., 2012). In general, positively charged particles show increased cellular uptake when compared to negative and neutral particles (Sahay et al., 2010), however, polyacrylamide nanosensors have a neutral zeta potential in biological conditions. Positively charged polyacrylamide sensors can be synthesized through polymerization of acrylamide monomers with charged monomers such as (3-acrylamidopropyl) trimethylammonium chloride (ACTA) (Figure 1iv). Silica sol-gel nanoparticles have a negative zeta potential, which is generally unfavorable for spontaneous cellular uptake, though can be useful when using cationic delivery vehicles such as Lipofectamine. Through substitution of the monomer N-trimethoxysilypropyl-N,N,N-trimethylammonium chloride (TMAC) into the sol-gel matrix (Figure 1viii), silica nanoparticles with a positive charge can be synthesized. Detailed methods for synthesis of charged polyacrylamide and silica nanosensors are described in this article.

It is important to note that amino-functionalized polyacrylamide particles have been shown to aggregate over time and as result possess a small window in which they can be used to conjugate secondary precursors (Welser et al., 2009). The shelf-life of the nanoparticles can be extended through replacement of amino groups with aizde and alkyne linkers. Azide and alkyne linkers are thought to be less susceptible to aggregation, because they mask the linker moiety, require an additional copper catalyst, tetrakis(acetonitrile)copper(I) hexafluorophosphate, and copper stabilizer tris-(benzyltriazolylmethyl)amine (TBTA), to become activated. This article will not explore methods to create azide and alkyne linker nanoparticles, however, further information can be found in an article published by Welser et al. (2009).

### Characterization

There are a number of well-established techniques that can be applied to characterize fluorescent nanosensors. Nanoparticles are typically characterized according to their size and surface characteristics using a series of complimentary techniques. Specialist techniques, solely for particle sizing, include dynamic light scattering (DLS) (Figures 2A,B) (Murdock et al., 2008) and disc centrifugation (Figures 2C,D). In addition, some of the principles of DLS are applied during zeta-sizing, which determines the zeta potential of nanoparticles. Complimentary microscopy and scanning probe techniques can yield information about the size and surface characteristics of particles, examples include (SEM) (Figures 2E,F) and atomic force microscopy (AFM) (Figures 2G,H).

### Calibration

Fluorescent nanosensors must be calibrated so that the response of the sensing element can be correlated to the concentration of the analyte of interest. Moreover it is important to establish the range in which the nanosensors are suitable for measurement.

The simplest approach to calibration is to suspend nanosensors in a range of buffer solutions of a pre-determined pH, where the pH of the solution is measured using a pH meter (Peng et al., 2007; Coupland et al., 2009; Benjaminsen et al., 2011). The drawback of this approach is that these conditions are very different to the conditions the sensor will experience in the cell. The most significant considerations are nanosensor concentration, ionic strength, and potential interference from biomolecules. A more representative calibration can be performed by conducting an *in situ* calibration. One approach is to immerse cells in buffer solutions and use ionophores to equilibrate intracellular pH with extracellular pH. Nigericin, which exchanges K^+^ for H^+^ ions, has been used for this purpose (Thomas et al., 1979). However, the efficiency of this relies on an even distribution of ionophores within the cell, which is unlikely particularly when sensors are held in internal organelles. An alternative method for calibration is proposed in this article based on controlling pH in fixed, permeabilized cells.

The relationship between the intensity and pH in the calibration is modeled by fitting an equation. This equation is subsequently rearranged to represent intensity as a function of pH values. In most cases there is a sigmoidal relationship between intensity and pH (Ruedas-Rama and Hall, 2006; Benjaminsen et al., 2011; Chauhan et al., 2011).

From a practical perspective, the instrument settings used to detect fluorescence should match those used in the measurement experiment as closely as possible.

### Image analysis

Image analysis is required to extract data from images. The method used for image analysis will affect final measurements, however, in many published articles the image analysis procedures are not stated in detail or not described at all (Burns et al., 2006b; Peng et al., 2007; Coupland et al., 2009; Ray et al., 2011). This could be a potential source for discrepancies in measurements reported in the literature. The main considerations for image analysis are (1) selecting the region within an image to be considered for analysis, (2) background removal, (3) automation of image processing and (4) presentation of data.

#### Measurement region

In most cases an image will contain dark regions where no nanosensors are detected; these regions must be excluded from the measurement. This is usually done by setting a threshold above which a region is considered to contain nanosensors. The region under consideration could be the entire image, individual pixels or discreet regions of interest (ROIs) in an image set by a size criteria (Christensen et al., 2002; Sonawane et al., 2002; Benjaminsen et al., 2011; Fares and van der Bliek, 2012; Chauhan et al., 2013). The advantage of taking the entire image is that it is possible to generate large amounts of data quickly, however, the disadvantage is that it gives no information about the distribution of intracellular pH. Conversely taking a pixel-by-pixel approach allows for a more detailed analysis however the computational time to process images is much larger. It is also requires pixels in corresponding color channels to be very accurately aligned; consequently this approach is more susceptible to errors in the instrumental setup. A ROI approach where the cell is considered as discrete regions is a compromise between the two approaches.

#### Background removal

Images acquired by microscopy and other fluorescence-based methods invariably contain background. The source of this background could be from cell autofluorescence, media fluorescence, or noise from the detector. Various methods for background subtraction have been proposed for conducting intracellular measurements. One approach is to take an image of the cell without any sensors, and approximate this to a mean value, which is subtracted from an image (Sonawane et al., 2003). In a similar method this value can be obtained from identifying an ROI outside the cell (Christensen et al., 2002). Other studies have approximated the background by analysing the frequency histogram of an image (Benjaminsen et al., 2011). However, most studies utilizing nanosensors do not include a detailed explanation of how background is removed from the images (Burns et al., 2006b; Peng et al., 2007; Coupland et al., 2009; Ray et al., 2011).

#### Automation of image analysis

Analysis of multiple images is required to extract representative data from images, however, this can be time consuming when working with large data sets. As performing ratiometric measurements with nanosensors is not currently routine, there are few examples of commercially available software with facilities for performing this type of analysis with control of variables of interest, therefore custom software solutions have been used in most studies. FIJI (open source) and MATLAB are widely available software solutions that can be tailored to perform ratiometric measurements.

#### Data presentation

It is important to consider how pH measurements from intracellular sensors are presented. Measurements have been reported as an average figure for an entire image or set of experiments (Coupland et al., 2009), or a histogram representing the distribution of pH values in an image or a color map showing discrete regions in a cell at a specific pH (Benjaminsen et al., 2011; Chauhan et al., 2013). Presenting images as a histogram or a color map has the added advantage of giving information about the distribution of pH within a cell. Additionally in an image there are always likely to be measurements, which are outside the range of the calibration curve. It is important that these pixels are represented.

### Perspective

**Figure 3:**
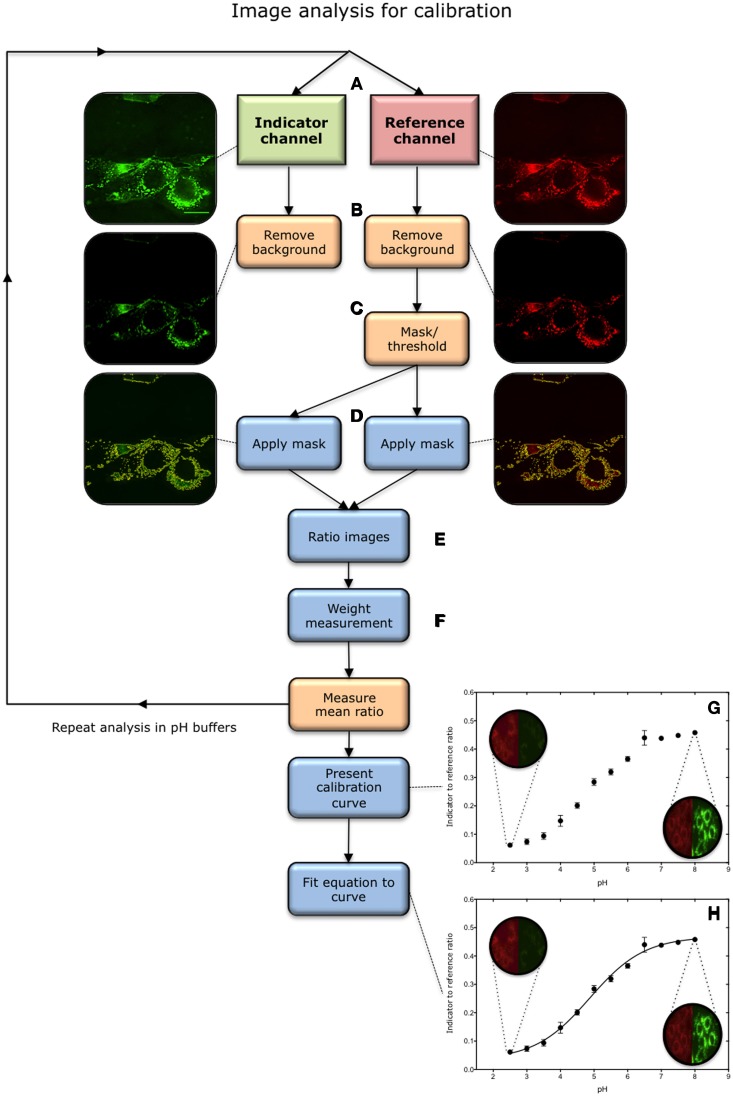
**Overview of image analysis process for calibration. (A)** Images are acquired in the indicator (green) and reference (red) channels in a universal buffer solution of known pH. The central most in-focus slice selected for analysis. **(B)** Background is removed in both channels. **(C)** Pixels containing nanosensor signal are isolated from the image. This is achieved by applying a threshold to the reference image, above which pixels are considered to contain nanosensors. This effectively creates a mask. **(D)** The mask is subsequently applied to the corresponding image in the indicator channel. **(E)** A ratio of indicator to reference intensity is taken for each pixel within the masked region. **(F)** The ratios are then weighted according to the intensity in the reference channel. **(G)** The process is repeated over a pH range from 2.5 to 8.0, and the mean intensity is utilized to construct a calibration. **(H)** An equation is then fitted to the plot.

**Figure 4:**
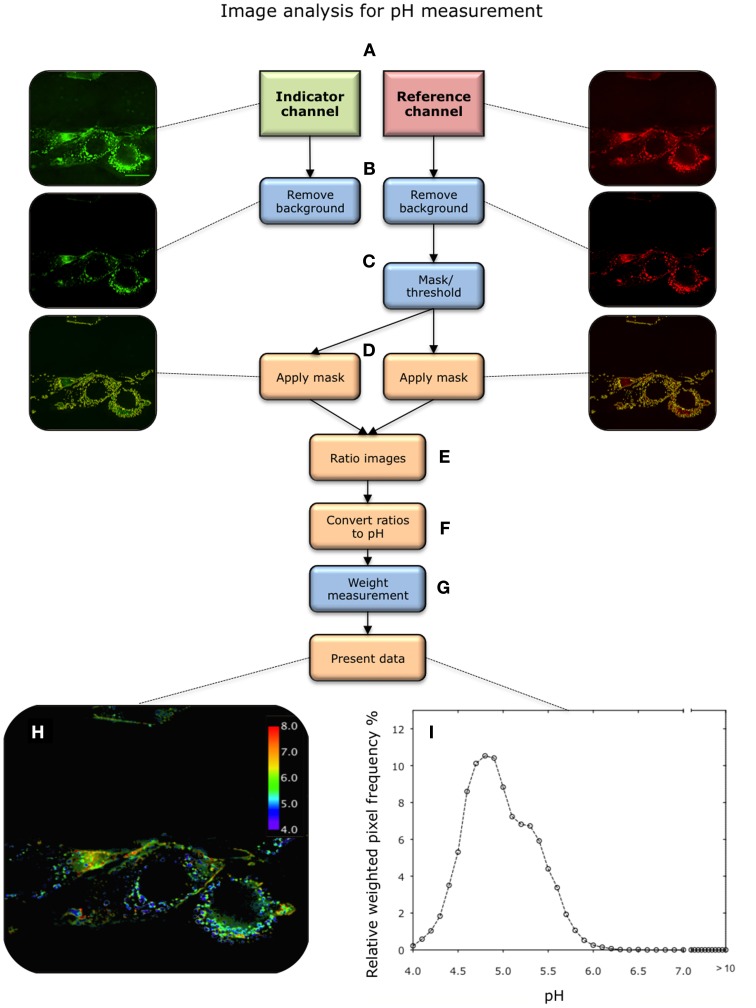
**Overview of image analysis procedure for pH measurement. (A)** Images are acquired in the indicator (green) and reference (red) channels after nanosensor uptake. The central most in-focus slice selected for analysis. **(B)** Background is removed in both channels. **(C)** Pixels containing nanosensor signal are isolated from the image. This is achieved by applying a threshold to the reference image, above which pixels are considered to contain nanosensors. This effectively creates a mask. **(D)** The mask is subsequently applied to the corresponding image in the indicator channel. **(E)** A ratio of indicator to reference intensity is taken for each pixel within the masked region. **(F)** This then converted to pH via the calibration curve. **(G)** The ratios are then weighted according to the intensity in the reference channel. **(H,I)** The image is then presented as a color map or a histogram.

**Figure 5:**
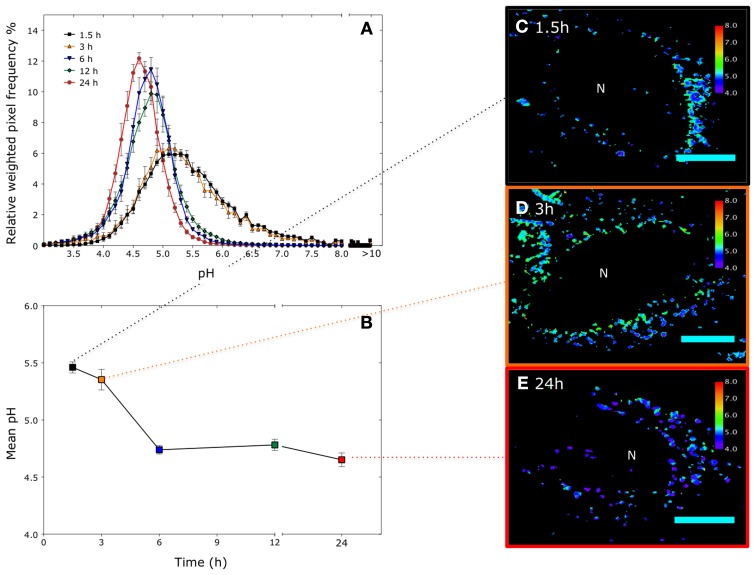
**Time resolved pH measurements in HeLa cells using a polyacrylamide nanosensors incorporating two pH-sensitive fluorophores and a reference fluorophore (5-(6)-carboxyfluorescein (FAM)/Oregon Green) and a reference fluorophore 5-(and-6)-carboxy- tetramethylrhodamine (TAMRA): (A) pH distribution in cells over different time points, pixels outside the calibration range are assigned pH > 10.** (*n* =~50 cells from 3 independent experiments, error bars represent *SD*). **(B)** Corresponding mean pH. (*n* =~50 cells, error bars represent *SD*). **(C–E)** Representative color mapped images at corresponding time points. Pixels outside the calibration range are represented as black pixels. Scale bar = 12 μm.

The development of fluorescent nanosensors has been taken on by a number of groups around the world. To date, fluorescent nanosensors have been reported to be sensitive to pH but also glucose, oxygen, calcium, zinc, magnesium, iron, adenosine triphosphate (ATP) concentration (Clark et al., 1999; Xu et al., 2001, 2002; Park et al., 2003; Sumner and Kopelman, 2005; Webster et al., 2007; Ozalp et al., 2010; Chauhan et al., 2011). The scope for producing new fluorescent nanosensors is limited only by the availability of indicator fluorophores. Comparatively the methodology for application of nanosensors is under developed. Further development is essential in order to pursue further application and also for making comparisons between different studies. This article contains detailed methodology for performing intracellular pH measurements using ratiometric polyacrylamide or silica nanosensors (Figures 3 and 4). The methods are annotated with notes outlining specific considerations when performing measurements and considerations for designing experiments using nanosensors. Finally we demonstrate the application of this method through temporal pH measurements in HeLa cells using polyacrylamide nanosensors (Figure 5).

## Materials

### Preparation of nanosensors

#### Polyacrylamide-based nanosensors

1. Solvents: Hexane, absolute ethanol.
2. Surfactants: Polyoxyethylene (4) lauryl ether (Brij30^®^), dioctyl sulfosuccinate sodium (AOT).
3. Monomers: Acrylamide, N,N methylene bisacrylamide (for synthesis of unfunctionalized nanosensors), N-(3-Aminopropyl)methacrylamide hydrochloride (APMA), (3-acrylamidopropyl) trimethylammonium (ACTA) (for synthesis of functionalized nanosensors).
4. Fluorophores: See Table 1 for a list of fluorophores.
5. Initiators/Catalyst: Ammonium persulfate (APS), N,N,N,N-tetramethylethylenediamine (TEMED).
6. Inert gas: Argon line at a pressure of 1 bar (14.5 PSI).

#### Silica-based nanosensors

1. Solvent: Absolute ethanol.
2. Monomers: Tetraethyl Orthosilicate (TEOS). (3-Amino propyl) trietoxysilane (APTES), methyl triethoxysilane (MTEOS) and 3-(trimethoxysilyl)propyl-N,N,N-trimethylammonium-N chloride (TMAC).
3. Catalyst: Ammonium hydroxide solution, 30% v/v in water.
4. Fluorophores: See Table 1 for a list of fluorophores.

#### Washing and filtration of nanosensors

1. Filtration: Glass microanalysis filter holder, filtering flask, vacuum pump 8 mbar and 0.02 μm pore, 25 mm polyamide filtration membrane.
2. Washing: Centrifuge.
3. Drying: Desiccator containing dried silica gel desiccant.

### Calibration

1. Preparation of cells: Paraformaldehyde 4% v/v in phosphate buffered saline (PBS). Triton X-100 1% v/v in PBS.
2. Universal buffer solutions: Sodium phosphate dibasic and citric acid monohydrate.

### Imaging and uptake

1. Cell culture: Serum and phenol red free cell culture growth media. PBS. Trypsin and ethylenediaminetatraacetic acid (EDTA) solution. Cell culture incubator maintained in a humidified atmosphere at 5% CO_2_ and 37°C^1^.
2. Imaging: Glass bottomed vessel suitable for microscopy and cell culture. Widefield or confocal microscope. See Note D1 for information on selecting microscopes.

### Image analysis

Software is required for image analysis. There are a number of packages available for automation of image analysis such as FIJI, MATLAB, Volocity, and Imaris.

## Methodology

### Synthesis of nanosensors

Fluorescent nanosensors are synthesized by incorporating analyte-sensitive and reference fluorophores into a nanoparticle matrix. Different combinations of fluorophores are used to tailor the sensor for a specific application. See Note D2 on selecting fluorophores for measurement. Generalized methods for the synthesis of nanosensors based on a polyacrylamide and sol-gel matrix are described here.

Procedures are performed at room temperature unless otherwise stated.

#### Preparation of fluorophores for incorporation into nanosensors

Fluorophores are incorporated into a nanosensor by entrapment or covalent attachment. Entrapment requires conjugation to dextran, whereas for covalent attachment, fluorophores are attached directly to a monomer. Fluorophore conjugates are incorporated into the nanosensor matrix during nanoparticle synthesis.

1. Conjugation of fluorophore to dextran: Dissolve 10 mg of 10,000 M.W. aminodextran in 5 ml of 50 mM sodium borate buffer pH 9. Add 0.05 mg of fluorophore. Add a magnetic stirrer bar and leave stirring for 2 h at room temperature followed by 12 h at 4°C.
2. Conjugation of fluorophore to APMA (for polyacrylamide nanosensors): Dissolve 5 mg of APMA in 2.5 ml of 50 mM sodium borate buffer pH 9 in a light protected glass scintillation vial with a magnetic stirrer bar. Take a 250 μl aliquot and dissolve 1 mg fluorophore in the solution. Leave stirring for 2 h at room temperature followed by 12 h at 4°C. Use 250 μl of this fluorophore-APMA solution for each batch of polyacrylamide nanosensors.
3. Conjugation of fluorophore to APTES (for silica sol-gel nanosensors): Dissolve 1 mg of fluorophore in 995 μl of anhydrous absolute ethanol in light protected scintillation vial purged with argon gas, to remove moisture. To this solution add 5 μl of APTES, and a magnetic stir bar. Purge the scintillation vial with argon gas and seal container. Allow the container to stir for 2 h at room temperature followed by 12 h at 4°C. Use 250 μl of this stock fluorophore-APTES solution for each batch of silica sol-gel nanosensors.

#### Synthesis of polyacrylamide nanosensors

1. Deoxygenate solvent: Place 200 ml of hexane on a stirrer plate and purge with argon for 30 min.
2. Prepare surfactant mixture: Weigh 1.59 g of AOT into a 100 ml round bottom flask. Add 3.08 g of Brij30^®^ to the same flask. Place a magnetic flea inside the flask and seal with a rubber bung. Secure the flask on a stirrer plate.
3. Deoxygenate surfactants: Run argon through the flask by inserting a needle connected to an argon line into the flask using a second needle as an outlet.
4. Prepare monomer solution: Weigh 540.0 mg of acrylamide and 160 mg of N,N methylene bisacrylamide into a 20 ml glass vial. Dissolve in 1.5 ml of water by sonication.
5. Add solvent: Add 42 ml of deoxygenated hexane to the round bottom flask and run Argon through for 5 min. Attach a balloon to the outlet needle to maintain an inert atmosphere. Remove the needle connected to the argon line after the balloon has been inflated (Note P1).
6. Add fluorophores: Mix monomer solution with 50 μl of dextran conjugated fluorophores or 250 μl of monomer conjugated fluorophores and make up to a final volume of 2 ml (Note P2). Add the monomer/fluorophore solution directly into the round bottom flask using a needle and syringe. (Leave for 10 min to allow the microemulsion to form).
7. Initiation: Weigh 100 mg of APS and leave on ice. When ready to initiate the polymerization add 1 ml of deionized water to APS to make a 10% w/v solution. Remove the balloon then quickly remove the bung and add 30 μl of solution immediately followed by 15 μl of TEMED. Reseal the flask and run argon through for 5 min. Reattach a balloon to the outlet needle. Remove the second needle after the balloon is inflated (Note P3).
8. Polymerization: Leave stirring for 2 h. Wrap the flask in foil if the fluorophores are light sensitive.
9. Termination: Stop the polymerization by removing the stopper.
10. Precipitation: Remove the hexane by rotary evaporation at room temperature. Continue evaporation until the solution becomes viscous and cloudy. Add 40 ml absolute ethanol to the round bottom flask and mix. Pour the mixture into a falcon tube. Centrifuge at 4000 rcf.
11. Washing: Pour off the supernatant and re-suspend the pellet in 40 ml of absolute ethanol. Centrifuge at 4000 rcf. Repeat 5 times. Re-suspend the pellet in the 10 ml of ethanol after the final wash.
12. Filtration and Drying: Filter the suspension through a 0.020 μm membrane filter using a vacuum filter until the particles appear dry. Collect the solid in a light protected vial, covered with a pierced film and place in a desiccator overnight to remove any remaining solvent (Note P4).
13. Storage: Seal the vial and store at −20°C.

Positively charged polyacrylamide nanosensors are prepared by substituting the monomer solution with the functionalization reagents found in Table 2.

**Table 2:** **Quantities of monomers required to synthesize blank, amine-functionalized, and positively charged nanosensors**.

**Functional group**	**Functionalization reagent**	**Monomers (mg)**		
		**Acrylamide**	**N,N methylene bisacrylamide**	**Functionalization reagent**
Blank	–	540.0	160.0	–
Amine	APMA	529.5	160.0	27.2
Positive	ACTA	513.2	154.2	78.5

APMA, N-(3-Aminopropyl)methacrylamide hydrochloride; ACTA, (3-acrylamidopropyl) trimethylammonium.

#### Synthesis of silica-based nanosensors

1. Prepare catalyst mixture: Using a magnetic stirrer and stir bar mix 5.5 ml of ethanol and 4 ml of ammonium hydroxide solution in a 50 ml round bottom flask, at 2000 rpm. Seal the flask with a stopper. Leave for 10 min.
2. Add Monomer: Add 500 μl of TEOS in a drop wise manner, at a rate of 50 μl/min, to the stirring catalyst mixture. Add 250 μl of monomer/fluorophore stock solution (Note P2).
3. A cloudy suspension should form on the addition of TEOS. Seal the round bottom flask with the stopper and stir for 1 h.
4. Washing: Add 30 ml of ethanol to the round bottom flask and transfer the suspension into a falcon tube. Centrifuge the falcon tube at 4000 rcf for 10 min. Carefully decant the supernatant and re-suspend the pellet in 40 ml of ethanol. Repeat this step 5 times. Re-suspend the pellet in 10 ml of ethanol after the final wash.
5. Filtration and Drying: Filter the suspension through a 0.020 μm membrane filter using a vacuum filter until the particles appear dry. Collect the solid in a light protected vial, covered with a pierced film and place in a desiccator overnight to remove any remaining solvent.
6. Storage: Seal the vial and store at −20°C.

Functionalized silica sol-gel nanoparticles are prepared by substituting the monomer solution with the functionalization reagents found in Table 3.

**Table 3:** **Quantities of reagents required to make blank, amine functionalized, hydrophobic and positively charged sol-gel nanoparticles**.

**Functional group**	**Functionalization reagent**	**Monomers (μl)**	
		**TEOS**	**Functionalization reagent**
Blank	–	500	–
Hydrophobic	MTEOS	500	477
Positive	TMAC (50%)	500	622
Amine	APTES	475	25

(3-Aminopropyl) trietoxysilane (APTES), Methyl triethoxysilane (MTEOS), and 3-(trimethoxysilyl)propyl-N,N,N-trimethylammonium-N chloride (TMAC).

### Nanosensor characterization

General sample preparation methods are given here. Readers should refer to manufactures protocols for detailed protocols.

1. Preparation of samples for Disc Centrifuge, DLS, Zetasizing, AFM: Re-suspend samples to a concentration of 5 mg ml^−1^ in filtered water. Use sonication if required.
2. Preparation of samples for SEM/TEM: Place a single droplet of 5 mg ml^−1^ nanosensors in filtered water onto a carbon coated electron stub and leave to dry overnight. Sputter coat the samples with gold for 4 min^2^ under Argon and image.

### Intracellular delivery of nanosensors

The procedure is described for positively charged nanosensors, but is applicable to all nanosensors, which do not require any additional methods to facilitate uptake.

#### Nanosensor uptake

Prior to uptake cells should be seeded into a vessel suitable for imaging on a fluorescence microscope. Suitable imaging vessels are glass-bottomed dishes and glass bottomed chambered cover glass. Cells should be cultured until 50–60% confluent in serum free media. The uptake experiment is then carried out as follows:

1. Cell uptake: Re-suspend nanosensors in PBS to a concentration of 2 mg ml^−1^. Sonicate until a clear solution is obtained (10–20 min). Replace cell growth media with fresh media and add sensors to a final concentration of 100 μg ml^−1^. Incubate at 37°C, 5% CO_2_ for desired time period.
2. Washing: Remove nanosensor containing growth media and wash cells by adding and removing fresh phenol red free growth media 3 times. Cells are now ready for imaging.

#### Image acquisition

Cells can be imaged by deconvolution wide field microscopy or confocal microscopy. Instrument settings should be kept consistent for uptake and calibration. The specific parameters are dependent on the application. Guidelines for setting instrument parameters are outlined below:

1. Pixel size: Set to the maximum theoretical resolution of the microscope with respect to the Nyquist criterion, if full resolving power is required.
2. Alignment: Test alignment by imaging multicolor fluorescent beads (TetraSpek^®^beads available from Invitrogen^®^can be used for this purpose). If there is any misalignment this should be corrected by performing a registration correction.
3. Bleed through: Test by imaging nanosensors labeled exclusively with either the reference or indicator fluorophore. If any bleed through is observed, the fluorophore combination should be reconsidered.
4. Light source fluctuations: Test by repeatedly imaging a single point in a fluorescent sample over 1–2 h. If significant fluctuations are observed, nanosensors should be recalibrated to ensure the excitation source is performing to specification.
5. Intensity of excitation light: For measurements utilizing a lamp as the source (wide field microscopy), set the exposure time to the minimum time required to provide an adequate signal to noise ratio (~1:5) without saturating the image. For microscopes using laser light sources (confocal microscopy), the laser power should be minimized with the same consideration. This is to minimize photobleaching and phototoxicity from prolonged exposure to fluorescent light.

### Calibration

The first stage of calibration is to prepare a series of universal buffer solutions.

1. Preparation of stock Sodium Phosphate Dibasic 0.2 M 250 ml stock solution: Weigh 7.098 g of sodium phosphate dibasic and place in a 250 ml volumetric flask. Add 200 ml of water to the volumetric flask and sonicate until all solid has dissolved. Make up the solution to 250 ml with deionized water and seal with a stopper. Invert the volumetric flask to ensure thorough mixing of contents.
2. Preparation of stock Citric Acid Monohydrate 0.1 M 250 ml stock solution: Weigh 5.254 g of citric acid monohydrate and place in a 250 ml volumetric flask. Add 200 ml of deionized water to the volumetric flask and sonicate until all solid has dissolved. Make up the solution to 250 ml with deionized water and seal with a stopper. Invert the volumetric flask to ensure thorough mixing of contents.
3. Preparation of pH buffer solutions, ranging between pH 2.5 and 8.0: Add the volumes of sodium phosphate dibasic 0.2 M and citric acid monohydrate 0.1 M (as described in Table 4), to 50 ml Centrifuge tubes. Seal centrifuge tube with cap, and vortex to ensure thorough mixing of contents. Use a calibrated pH meter to record the pH of the buffer solutions. Aliquots of these solutions can then be used to calibrate nanosensors.

**Table 4:** **Volumes of sodium phosphate dibasic 0.2 M and citric acid monohydrate 0.1 M required to make pH buffer solutions from pH 2.5 and 8.0**.

**pH**	**Volume (ml)**	
	**Sodium phosphate dibasic (0.2 M)**	**Citric acid monohydrate (0.1 M)**
2.5	2.16	17.84
3.0	4.08	15.92
3.5	6.04	13.96
4.0	7.72	12.28
4.5	9.00	11.00
5.0	10.28	9.72
5.5	11.36	8.64
6.0	12.84	7.16
6.5	14.20	5.80
7.0	17.44	2.56
7.5	17.98	2.02
8.0	19.53	0.47

Two different methods for calibrating nanosensors are described here firstly a cell free calibration and an *in situ* calibration. Buffer calibration is faster than the *in situ* calibration but less accurate for intracellular measurements.

#### Cell-free calibration

1. Preparation of a nanosensor suspension: Re-suspend nanosensors to a concentration of 10 mg ml^−1^ in PBS. Vortex or sonicate, until a clear solution is seen.
2. Suspend nanosensors in buffers: drop 45 μl of universal buffer solution on to a microscope slide followed by 5 μl of nanosensors resulting in a final concentration of 1 mg ml^−1^. Acquire images from a minimum of 5 different regions.

#### *In-situ* calibration

1. Cell uptake: Perform cell uptake procedure described in section Nanosensor Uptake (Note D3).
2. Cell fixation and permeabilization: Immerse cells in 4% paraformaldehyde in PBS solution for 15 min. Remove paraformaldehyde and immerse in 1% v/v Triton X-100 solution in PBS. Leave for 10 min at room temperature.
3. Acquire images for calibration: Remove Triton X-100 and immerse cells in buffer solutions from pH 2.5 to 8.0. Acquire images in a minimum of 5 different regions.

Image acquisition settings for calibration should be identical to those used for nanosensor uptake.

### Image analysis

#### Calibration

The first stage of the process is to analyse calibration images in order to generate a calibration curve. The process is described for *in-situ* calibration based on a pixel-by-pixel analysis. The procedure is summarized in Figure 3.

1. Remove background from images: Select a ROI outside the cell and subtract the value from the image. It is equally valid to obtain the background value from imaging cells without any sensors.
2. Identify nanosensor-containing pixels: Set a threshold, above which pixels are considered to contain nanosensors. This effectively creates a mask, which is subsequently applied to the corresponding image in the indicator channel. The threshold can be set subjectively as it will not have a great impact on calibration^3^.
3. Ratio images: Measure the indicator to reference ratio in each pixel within the masked region.
4. Weight measurements: Assign a weight to the measurement registered in each pixel based on the intensity in the reference channel, i.e., the concentration of nanosensor in each pixel (Note P6).
5. Construct a calibration curve: Repeat steps 1–4 for all images acquired across the pH range from 2.5 to 8. Use the mean ratios to construct a calibration curve.
6. Model calibration: Fit a sigmoidal curve to the calibration points. Using the equation:
  $$R_{i}=R_{\min}+\frac{R_{\max}-R_{\min}}{1+10^{(\text{p}Ka-\text{pH}).\text{ hillslope}}}$$
  Where:
  *R*_*i*_ = indicator to reference ratio
  *R*_min_ = Minimum detectable nanosensors response (lower asymptote)
  *R*_max_ = Maximum detectable nanosensors response (upper asymptote)
  p*Ka* = Point at half maximum response
  hillslope = Steepness of the curve
7. Rearrange the calibration curve to represent pH as a function of intensity.

$$\text{pH}=-\frac{\log_{10}\left(\frac{R_{\max}-R_{\min}}{R_{i}-R_{\min}}-1\right)}{\text{hillslope}}+\text{p}Ka$$

This equation is used to calculate pH from nanosensor uptake images.

#### pH measurements

The image analysis process follows the same process as for calibration to generate a ratiometric image. The process is summarized in Figure 4. Thresholding at this stage of analysis has an effect on final measurement, therefore consideration should be give to how this is set (see Note P5). After this the following procedure is followed:

1. Convert ratios to pH: Convert each pixel a pH value using the equation generated from the calibration curve.
2. Weight measurements: Assign a weight to each pixel pH based on the intensity in the reference channel, i.e., the number of nanosensors in each pixel (Note P6).
3. Present data in a histogram: Bin the data to plot the measurements as a histogram. Any pixels reporting pH outside the range of the sensor should be accounted for.
4. Present data as a color map: Color each pixel on a linear scale.

## Notes

Notes annotated with “P” are specific practical considerations whilst performing experiments. Notes annotated with “D” are considerations when designing experiments using nanosensors.

### Note P1

The solvent to monomer ratio is critical to the reaction. Ensure Argon is run through the monomer/solvent solution for a maximum of 5 min to prevent evaporation which could alter the ratio.

### Note P2

The amount of fluorophore to be added is dependent on the required brightness of the nanosensors for the chosen application, and the brightness of the fluorophore in use. Typically between 25 and 250 μl of stock solution are used, however, optimization may be required for the specific application.

### Note P3

It is important to add the initiators to the monomer/solvent solution quickly to prevent termination of the reaction by oxygen. APS must be made freshly for each batch of nanosensors.

### Note P4

Alternative methods for drying the nanosensors are rotary evaporation, purging with argon and storage in a desiccator. These methods can be used in combination with vacuum filtration. We have found vacuum filtration to be the most reliable method for drying nanosensors.

### Note P5

The threshold should be set at the lowest value where there are more than 90% of pixels in the range of the calibration curve. This can be determined by recording the error across a range of thresholds in a test image for a given experiment. This is important because a very high threshold will lead to the exclusion of data, whereas a threshold which is too low is likely to result in a high proportion of pixels outside the calibration range.

### Note P6

Weighting is an additional processing step incorporated to increase reliability of measurements. In the case of an unweighted image, each pixel is assigned a pH value, which is represented in the histogram. The problem with this is that a pixel is represented as one unit on the histogram whether it has a very intense or very weak signal. However, it is apparent that nanosensors are concentrated within discrete areas of the cell. In order to correct for this, pixels are weighted using the intensity of the reference image. Weighting is essential to determine the proportion of sensors which are reporting a pH, this is important as without this measurement of pH would merely be an indicator of the spatial distribution of pH inside a cell.

### Note D1

Confocal microscopy is the overwhelming method of choice for conducting intracellular measurements. This is because it provides a higher resolution than conventional wide field systems and more reliable results from elimination of out of focus light. Confocal microscopy is now a mature technology, however there are a number of disadvantages relative to wide field systems. High power lasers induce phototoxicity and as most of the light is removed by the pinhole so very bright specimens are required for imaging. Conventional wide field systems, although useful at low resolutions, produce too much out of focus light to produce reliable measurements from nanosensors at high resolutions. It is possible to increase the resolution of wide field techniques using a post-processing techniques such as deconvolution.

Deconvolution is a well-established technique for improving the contrast and resolution of an image by removing or reassigning out of focus light or blur. Blur arises from the spreading of light (diffraction), which occurs as light passes through the optical train of the microscope before reaching the detector. The way in which the light is diffracted is a function of the components of the microscope, principally the objective. Therefore, it is possible to mathematically model the blur and remove or reassign it from an image. As all optical systems produce blur, it is possible to use deconvolution on different types of microscopy techniques including confocal microscopy. However, it is a particularly powerful technique for wide field systems producing dramatic improvements in image quality.

In summary, confocal microscopy is the method of choice if high-resolution images are required, providing bright samples are available and cells are sufficiently insensitive to photo damage. Deconvolution techniques are developing quickly therefore wide field techniques may become the method of choice in the future.

### Note D2

The key consideration for selecting fluorophores for intracellular measurements is the range and sensitivity of the fluorophores. Incorporating a combination of fluorophores can be used to extend the range of sensors as demonstrated in our previous work (Chauhan et al., 2011). In this study two pH-sensitive fluorophores [fluorescein isothiocyanate dextran (FITC-D) and Oregon Green dextran (OG-D)] and a reference fluorophore (5-(and-6)-carboxy—tetramethylrhodamine dextran (TAMRA-D)) were incorporated into a single sensor resulting in a dynamic range from pH 4.0 to 7.5. This covers the expected intracellular pH range.

### Note D3

The concentration of nanosensors and length of exposure can be altered if inadequate uptake is observed. If higher nanosensor concentrations are required it is advisable to test the toxicity of the sensors. We have observed no toxicity in an MTS assay for cell proliferation up to concentrations of 2 mg ml^−1^ in 3t3 and MRC-5 fibroblast cells.

## Funding

This work is supported by an industrial CASE studentship (Veeren M. Chauhan) from the Biotechnology and Biological Research Council (BBSRC) and a Doctoral Training Centre studentship (Arpan S. Desai) supported by the Engineering and Physical Sciences Research Council (EPSRC) and AstraZeneca (AZ) Grant number: EP/D501849/1.

### Conflict of interest statement

The authors declare that the research was conducted in the absence of any commercial or financial relationships that could be construed as a potential conflict of interest.

## Footnotes

^1^Formulation of growth media, trypsin, and growth conditions is dependent on the cell type.

^2^Sputter times are dependent on the instrument.

^3^A thresholding step is not required for cell free calibration because nanosensors will distribute evenly across the field of view.
